# Unintended Changes of Ion-Selective Membranes Composition—Origin and Effect on Analytical Performance

**DOI:** 10.3390/membranes10100266

**Published:** 2020-09-28

**Authors:** Krzysztof Maksymiuk, Emilia Stelmach, Agata Michalska

**Affiliations:** Faculty of Chemistry, University of Warsaw, Pasteura 1, 02-093 Warsaw, Poland; kmaks@chem.uw.edu.pl (K.M.); ewoznica@chem.uw.edu.pl (E.S.)

**Keywords:** ion-selective membranes, components leakage, incorporation, all-solid-state sensors

## Abstract

Ion-selective membranes, as used in potentiometric sensors, are mixtures of a few important constituents in a carefully balanced proportion. The changes of composition of the ion-selective membrane, both qualitative and quantitative, affect the analytical performance of sensors. Different constructions and materials applied to improve sensors result in specific conditions of membrane formation, in consequence, potentially can result in uncontrolled modification of the membrane composition. Clearly, these effects need to be considered, especially if preparation of miniaturized, potentially disposable internal-solution free sensors is considered. Furthermore, membrane composition changes can occur during the normal operation of sensors—accumulation of species as well as release need to be taken into account, regardless of the construction of sensors used. Issues related to spontaneous changes of membrane composition that can occur during sensor construction, pre-treatment and their operation, seem to be underestimated in the subject literature. The aim of this work is to summarize available data related to potentiometric sensors and highlight the effects that can potentially be important also for other sensors using ion-selective membranes, e.g., optodes or voltammetric sensors.

## 1. Introduction

Ion-selective membranes (ISMs) are used in different configurations and in various types of sensors ranging from electrochemical: potentiometric, voltammetric/coulometric, electrolyte gated transistors to optical sensors. An ISM as understood here is a system as typically used in potentiometric sensors: a membrane containing ionophore—a ligand able to preferentially bind analyte ions (called primary ions) in the lipophilic medium [1,2]. Due to the presence of a highly selective ionophore, the ISM allows determination of contents of free ions of interest, in the presence of other chemical forms of analyte, in complex matrices, including blood, serum or environmental samples [3,4,5,6,7,8]. This makes ISM-based sensors attractive tools for many applications. ISMs are used in various sensor constructions: classical size (macroscopic) and those belonging to nanoscale, intended for disposable use or for long-term operation, containing internal solution or using alternative constructions. Taking into account envisaged applications it is required that sensors are characterized with a high stability of performance, including sensitivity, selectivity as well as stability and reproducibility of potential reading in time [9,10,11]. It is also advantageous, if the sensor construction allows miniaturization and mass-scale production of devices. The envisaged disposable/in-fields operation sets additional demands such as high reproducibility between different sensors from a production batch, potentially allowing calibration-less operation [12,13,14,15].

Classical operation mode of ISM-based sensors is equilibrium mode, ion-exchange occurring between the membrane and the sample is driven by the preference of analyte ions in the ISM phase (typically achieved due to complexation with ionophore) [1,2]; although electrochemical trigger applications (non-equilibrium mode) of ISMs have been also proposed [16]. It is generally accepted that optimal analytical performance of the membrane requires that composition of the phase is well defined by the application of tailored amounts of the defined components during preparation. It is assumed that the intended composition of the membrane is maintained through the sensor’s lifetime, with only one exception. In most of cases incorporation of the primary ions (to as prepared membrane) in the pre-treatment step occurs, which is needed to assure stable performance of the sensor.

**Figure 1:**
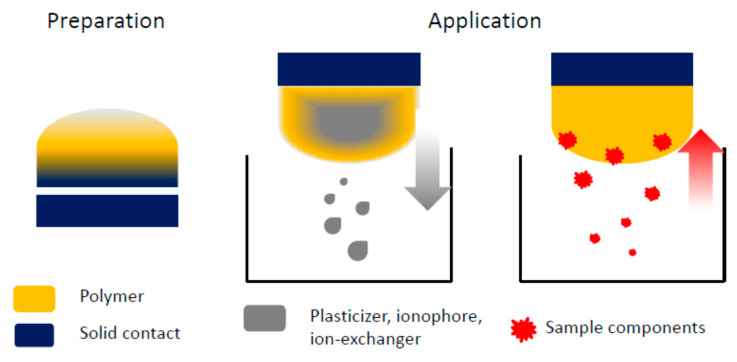
Schematic representation of processes related to ion-selective membrane occurring during sensor preparation as well as application.

However, during preparation of potentiometric sensors of different constructions, there are diverse spontaneous processes occurring through the sensor’s operation, Figure 1, that may affect analytical responses, the lifetime of the device, or its application safety. Their occurrence can be obscured, and resulting changes can be difficult to trace, leading to variation of performance, that can be attributed to various effects. These processes are generally off the main stream of ISM sensors research, the major focus in the field being on improving performance of the devices. In this work we intend to highlight the processes that can affect ISMs potentiometric sensors operation, and potentially need to be considered while aiming construction improvements, application of new materials etc. It is also shown that considering spontaneous changes and their effect can help to improve sensors, to minimize adverse effects.

## 2. Ion-Selective Membranes (ISM)

Ion-selective electrodes (ISEs) with polymeric ion-selective membranes have been studied and used for about 50 years [17,18,19,20,21,22]. The analytical performance of ISMs containing sensors—slope of dependence, linear range of responses, selectivity, are mostly dependent on properties of the ion-selective membrane [1,2,23]. The composition of ISMs is carefully balanced—to contain just the intended compounds of high purity, in the right proportions. Typically, an ISM comprises: ionophore, cation-exchanger, polymer matrix (polymer optionally with plasticizer) [1,2]. Classic composition of an ion-selective membrane is a few *w*/*w*% (3–5) of each: ionophore and ion-exchanger, and the rest of the membrane mass is the polymer matrix. Typically, the amount of ion-exchanger used is about 60% of the mole amount of ionophore used, thus the presence of excess of free ionophore in the ISM is assured [1,2]. The polymer matrix is mixed with ionophore and ion-exchanger in volatile (auxiliary) solvent, commonly tetrahydrofuran (THF), and applied from dispersion—a membrane cocktail. Spontaneous evaporation of the auxiliary solvent, in the laboratory atmosphere, results in the formation of a polymer layer— the ISM. The membrane can be formed on an inert surface (e.g., glass) and then transferred to the sensor or it can be obtained from a cocktail (solution of membrane components) by drop casting on the top of the transducer of choice to form the sensor. Alternatively, other methods of membrane deposition from a cocktail, e.g., spraying, can be used [24]. If the membrane polymer requires the presence of a plasticizer, which is the most typical case for, e.g., poly(vinyl chloride) (PVC), the compound—liquid used—remains in the formed ISMs (by contrast with THF). For PVC-based systems, plasticizer is relatively abundant in the membrane—its content is typically close to 60% *w*/*w* of the film formed.

Some polymeric membranes, e.g., polyacrylates, are formed in a polymerization process from cocktails containing ionophore, ion-exchanger and monomer together with e.g., the cross-linker and polymerization initiator dissolved in monomer solution [25,26,27]. Under e.g., ultraviolet (UV) irradiation, polymerization of the monomer occurs resulting in film formation. This method of membrane formulation, allowing elimination of liquid plasticizer and auxiliary solvent, seems to be an attractive alternative to avoid problems encountered with classic liquid cocktails of e.g., PVC-based ISMs. Despite the liquid monomers used, the amount of liquid cocktail used is significantly reduced and the application of very good solvent such as THF is eliminated, e.g., [25,26,27].

It should be stressed that in most cases the ISM is just a physical mixture of components [1,2]. For macroscopic potentiometric-type sensors, the thickness of the membrane is around 150–200 μm, and the membrane is a relatively lipophilic film of a few mm in diameter (typically 5–7 mm), that is in direct contact with the sample at one side, and on the back side it is in contact with the internal solution or solid contact (ion-to-electron transducer) material.

Although the composition of an ISM is precisely controlled during cocktail preparation, formation of the film and its application allows the occurrence of spontaneous processes resulting in change of the membrane composition, ultimately affecting the analytical performance of the sensors. Apart from primary ions exchange with the solution, other components of the ISM and/or solution, can be exchanged across the membrane interfaces: one with the sample or with the internal solution (IS)/solid contact (SC), depending on the construction applied. It can be expected that changes occurring can be driven by the solubility or partition coefficient of the involved species, both in the aqueous phase of the sample and in the organic phase of the membrane during operation and preparation. Leakage of components from the membrane as well as incorporation to the ISM phase can occur, depending on the sample nature, membrane, optionally SC or prevailing conditions. These processes can be related to ISM formation, pre-treatment and operation; being dependent on the construction of the sensor.

Spontaneous processes related to the ISMs operation are rarely considered. The presented reports on spontaneous changes of the composition of ISMs are related to potentiometric sensors mostly; however, it seems rational that these processes will equally involve other systems using similar compositions as ion-selective membranes of voltammetric or optical sensors. Leakage of components from the membrane is increasing in importance if the application of sensors in contact with humans is considered (e.g., wearable sensors, implantable devices) due to related health risks [28]. Moreover, spontaneous changes of ISMs can increase in importance if the volume of the phase is reduced as in the case of membranes of reduced thickness [29] or increased surface to volume ratio, as for e.g., nanospheres optical sensors [30].

In this work we focus on spontaneous effects related to potentiometric sensors ISMs—ion-selective electrodes that are intended to operate in equilibrium conditions. The aim of this work is to highlight the effect of spontaneous processes leading to changes in the composition of ISMs and the potential influence of spontaneous effects on the performance of resulting sensors.

## 3. Construction of Sensors

The overall performance of ISM-based potentiometric sensors is affected jointly by construction and membrane properties. It is generally assumed that stability of potential readings in time is mainly affected by construction of the sensor [31,32,33,34], whereas analytical performance is determined by membrane properties [1,2]. A classic ISM-containing arrangement offers high stability of potential readings in time due to well defined, reversible ion/electron transfer through all sensor interfaces. From the point of view of spontaneous processes related to the ISM this system is clearly less affected by the sensor preparation step. ISMs membranes intended for applications in internal solution ion-selective sensors are usually prepared by applying a membrane components solution—cocktail—to an inert material mould, thus even large quantities of THF used do not lead to accumulation of other substances in the PVC film formed. After solvent evaporation, individual membranes are cut off from the resulting layer and mounted in the sensor housing. Moreover, the internal solution arrangement is typically of well-defined composition and has limited volume (not exceeding 1 mL).

For ISEs, however, miniaturization/mass-scale production is often difficult, thus the solid contact (SC) construction was proposed [31,32,35,36,37]. The SC arrangement takes advantage of the presence of an ion-to-electron transducer layer between the electron conducting substrate electrode and the ISM. In the SC arrangement, the membrane is in contact with the solid material of various nature/chemical properties, and prepared using different methods [32]. Application of the membrane to make SC-type sensors is principally different from preparation of the ISM for ISE. Due to the variety of materials applied as SC, different conditions prevail during sensor construction. To obtain a membrane, typically an ISM cocktail is drop cast on top of the formed SC—the transducer layer. In this process an auxiliary solvent, e.g., a THF-based solution of the ISM cocktail is in contact with the transducer layer for minutes or hours before the solvent is evaporated. The amount of cocktail applied is dependent on *w*/*w* concentration of membrane components and the required thickness of the ISM. Typically, 20–30 ul of membrane cocktail is applied on one substrate electrode of diameter 3 mm [13]. If SC contains material (either main component, additive or impurity) soluble in the cocktail solvent, application of the membrane can result in partition of some of the transducer components to the ISM phase.

Various transducer systems have been proposed, including silver complexes [38], hydrogels [26], redox polymers [39]. For many years conducting polymers (CPs) have remained one of the most popular transducer materials; a wide range of CPs has been tested—from relatively hydrophilic electropolymerized materials like oxidized polypyrrole [27,40,41] or polyaniline [42] to more hydrophobic solvent processable CPs. Among hydrophobic polymers, alkylpolythiohenes, e.g., poly(3-octylthiophene) [43] or composites [44] render hydrophobicity due to the dopant/component applied [45,46], or other systems [47,48]. Although the majority of SC systems proposed use plasticized PVC-based ISMs, other membrane materials. e.g., polyacrylates [27,49] were also successful in this construction.

Electropolymerization of CPs was especially popular in the early years of conducting polymer-based SC systems [31,32]. The benefit of this method of SC formation is well-controlled composition of the formed layer—polymer typically with doping anions [31,32]. This method has been applied for many CP systems that are not solution processable. Typically, highly oxidized and conducting films formed by electropolymerization tend to undergo spontaneous discharge to a more stable oxidized state, and this process is related to exchange of ions with the solution e.g., [50,51,52]. If discharge of the CP transducer occurs through the ISM, this process can lead to pronounced accumulation of ions at the back side of the membrane, depending on ion-exchange properties of both ISM and transducer layer [53]. Equilibration of the CP before covering with the ISM, although it makes sensor preparation longer [54], helps to avoid excessive ion exchange between the transducer and the membrane, and their consequences such as electrolyte ions accumulation.

It should be stressed that most of transducer layers nowadays are obtained using dispersions e.g., of solvent processable CPs e.g., [55,56,57]. The transducer material in form of dispersion is e.g., drop cast on the substrate electrode surface, and after evaporation of the solvent it is covered with ISM (in the process similar to that used for electropolymerized SC).

One of the first solution processable materials used as SC were CPs prepared in the presence of a surfactant such as doping anions [58], especially poly(3,4-ethylenedioxythiophene) doped with poly(4-styrenesulfonate) ions (PEDOT–PSS) [55,56], a transducer already successful as a SC when obtained by electropolymerization [59]. The potential adverse effect of surfactant poly(4-styrenesulfonate) present in the SC was not observed, which can be attributed to its interaction with ions—precipitate formation within the SC, before or after membrane formation, thus preventing its partition to the membrane [56,58].

One of the most popular CPs is polyoctylthiophene (POT) e.g., [57,60,61,62,63,64,65,66]. Among advantages of polyalkylthiophenes are solution processability, high lipophilicity and low ions contents in the semiconducting state, which results in a low ion-exchange rate between the SC and ISM. Moreover, POT is soluble in various organic solvents without need of application of surfactants. The possibility of using solution processable materials as solid contacts offers significant advantages in terms of sensor construction, however, it brings significant risk of unwanted, uncontrolled transfer of SC material to the ISM phase, effect that was already mentioned for electropolymerized POT [67,68]. The magnitude of this effect for application of solution-processable POT as transducer is increasing [69]. Application of an ISM cocktail on a formed POT transducer layer results in visible change of the membrane colour and ultimately in CP presence in the membrane in amount close to that of ion-exchanger purposely added, i.e., ca 0.5% *w*/*w* [69]. Such high contents of conducting polymer can lead to disturbance in potentiometric responses of the phase, and it needs to be stressed that for sensors based on CP mixed with ionophore, ion-exchanger receptor layers have been proposed previously [70,71].

The other successful group of transducer materials are carbon-based nanostructures: reduced graphene oxide, graphite, macroporous carbon [46,72,73,74,75,76,77] and especially carbon nanotubes (CNTs) [24,78,79,80]. CNTs, similar to POT, are commercially available and can be prepared as dispersion in solution, allowing application by drop casting [24,78,79,81] or spraying [24]. Sensors using carbon nanostructures as transducer materials are prepared in different variations: using glassy carbon electrodes support, but also on plastic [24,81], or paper [14,82,83,84]. On the contrary to POT, CNTs are typically applied from dispersions stabilized with surfactant solution, e.g., sodium dodecyl sulfate. In early works of Rius [78,79] about the post-formation of the SC layer, the transducer was washed with water to remove excess of the surfactant. However, the control of effectiveness of this process is not precise. The presence of surfactants affects significantly wettability of CNTs [80,83]. Highly dispersible in water, CNTs containing surfactants are characterized with high hydrophilicity, as estimated using water contact angle [80,83]. Moreover, the presence of unbound surfactants in the transducer layer brings a risk of partition of these compounds to the lipophilic membrane phase, and this effect has been observed previously for samples containing surfactants [85,86,87]. This in turn can result in impaired performance of the ISM as well as a change of the properties of the transducer and loss of adhesion of membrane phases and ultimately sensor failure [80]. An alternative is dispersing CNTs using other agents, such as POT offering high capacitance of the transducer and high lipophilicity [44], or carboxymethyl cellulose as water-based dispersion. In this case high stability of potentials of prepared sensors was observed [80].

Partition of the transducer material to ion-selective membranes in the course of sensor preparation has been also reported for other systems. Application of SC based on CNTs containing porphyrinoids resulted in spontaneous partition of the latter to the membrane phase [13]. The higher loading of SC with porphyrinoids resulted in higher contents of these in the membrane [13]. It was also clearly confirmed that the presence of porphyrinoids affects performance of the sensor resulting in tailoring fluxes in the ISMs phase and ultimately leading to improved detection limit and selectivity [13].

## 4. Pretreatment and Operation

Typically, as-prepared ISMs do not contain primary ions and are not equilibrated with an aqueous phase. The only exception is using an ion-exchanger containing a counter ion, the membrane primary ion, e.g., in the case of using potassium salt of cation-exchanger to prepare potassium sensors, sodium or calcium salts to prepare respective sensors [62]. However, even in these cases the prepared ISMs are not equilibrated with water phase, thus fluctuation of recorded potentials can be observed directly after immersing in the sample solution [88]. Pretreatment of the ISM phase results in significant changes in composition of the membrane phase [89], which affects performance of the sensor and ISM analytical parameters [90]. The construction of the sensor applied (IS, or SC) affects this process—SC is typically a more complicated system than IS. For SC, the effect of supporting electrode material as well as properties of the transducer need to be taken into account, also in this step [61,88]. Despite this, some of the processes occurring are similar for IS and SC type sensor.

ISM pretreatment requires analyte ions transfer through the interface between the membrane and solution, formation of a complex with ligand (ionophore) in the membrane, and transport of the formed complex through the membrane. These processes are accompanied by expulsion of ion-exchanger counter ions from the membrane. Depending on composition of the sample and internal solution/solid contact, gradients of ions are formed in the membrane [61,89,91] and ultimately the ion content of the membrane changes. Depending on required sensor performance, pretreatment of ISM can result in different contents of primary ions in the membrane. Sensors intended to show low detection limits [92,93] need to have tailored fluxes of primary and interfering ions in the ISMs phase [94], regardless of construction applied. From a technical/construction point of view, it is typically easier to achieve this for IS type sensors [62,95,96].

For sensors intended to show classical detection limits close to 10^−6^ M, the time needed to spontaneously equilibrate ISMs with solution is dependent on the availability of primary ions in solution and transport of ions in the membrane phase [97,98]. For low sample concentrations (<10^−4^ M) transport of ions in solution becomes the rate-limiting step in the whole equilibration process [99]. However, if the concentration of primary ions in the solution is higher (>10^−3^ M), the rate-limiting step in equilibration of the membrane with primary ions is transport of ions in the membrane phase. Diffusion coefficients of ions in the membrane phase are typically much lower compared to ion-diffusion coefficients in aqueous solution [47,49,89]. Assuming typical thickness of the membrane in the range of 200 μm and diffusion coefficient in the membrane close to 10^−8^ cm^2^/s—characteristic for plasticized PVC [89]—the equilibration time needed for high concentration of primary ions in solution is under 12 h. The resulting levels of primary ions are comparable with the ion-exchanger (mole) amount added to the membrane cocktail [97,100]. It should be stressed that the diffusion coefficient obtained for polyacrylate polymers is much lower, close to 10^−11^ cm^2^/s, making equilibration with the solution a long-term process [49]. In the case of these materials spontaneous, extremely slow transport of ions within the membrane phase affects significantly the performance of potentiometric sensors [101]. A similar effect, however, in the case of optical sensors results in a significantly increased response range covering even 8 orders of magnitude [102], and offers linear dependence of the signal on logarithm of analyte concentration changes in the sample [71]. One of the possible options to affect (usually slow) ion-diffusion in the ion-selective membrane phase is elimination of the polymer, e.g., using thin liquid layers supported on inert material to host ionophore and ion-exchanger [103,104].

On the other hand, pretreatment of an SC-type sensor can lead also to an ion-exchange process occurring between the membrane and transducer, resulting in a change in composition of this layer. Changes in SC contents have been reported especially for layers originally rich in ions such as dispersion of CPs [61], highly oxidized CPs [53]. This process can lead to advantageous properties of the sensor, e.g., due to binding primary ions within the SC phase coupled with release of interfering ions initially present in SC [58]. It should be stressed that the absolute amount of material used is also important in this respect. Typically, SC contact contains a smaller absolute amount of ions compared to IS, thus it offers limited possibilities of maintaining in the long term the desired ion fluxes through the ISMs. On the other hand, increase of amount of transducer material used to prepare sensors can result in SC being a rich reservoir of ions and ultimately result in unexpected change of performance of the sensor simply due to the change of scale. For example, change of the sensor arrangement from glassy carbon substrate covered with CNTs dispersion to paper type sensors—requiring making conductive paper by application of the CNTs dispersion but in larger quantities—results in alteration of ISE performance. Due to increased contents of interfering ions (originating from dispersion) at the back side of the ISM pronounced ion exchange between the transducer and the membrane is induced [14].

The other issue related to ion-exchange between the ISM and solution is excessive incorporation of primary ions, or coextraction of solution ions. This leads to permselectivity failure [105,106], which is observed mostly in the case of lipophilic ions present in solution as an upper detection limit, or in extreme change of the dependence type from cationic to anionic [107]. The occurrence of these effects has been observed using a potentiometric approach, but also confirmed using spectropotentiometry [108,109] or membrane contents quantification [97,100]. The latter has shown 6 or 8 times higher (mole) amount of primary ions compared to ion-exchanger present in the membrane, for plasticized PVC or polyacrylate more-lipophilic membrane, respectively [100].

It should be stressed that ISMs able to preferentially bind primary ions can lead to accumulation of these ions from the sample, even if the analyte is at the impurities level in the presence of significant excess of other interfering ions [89]. This effect of unintended saturation with primary ions is not readily manifested unless the composition of the membrane is verified with e.g., inductively coupled plasma–mass spectrometry (ICP-MS), as observed in the presence of traces of lead(II) ions (4·10^−7^ M) in 0.1 M NaCl solution resulting in accumulation of primary ions in lead-selective membrane [89].

The presence of relatively lipophilic compounds as surfactants, regardless of their charge, in the sample solution, results in deterioration of analytical performance of ISM sensors [85,86,87]. This effect is attributed to the partition of lipophilic molecules to the membrane phase. Although it was studied for samples and IS-type sensors [85,86,87], as pointed above this can be observed for SC type sensors, if the SC can be source of surfactants. Presence of lipids in the sample also results in unwanted accumulation of these in the ISM leading to deterioration of performance over time [110].

It has been documented by many reports that pretreatment of the membrane is also connected with incorporation of water to the membrane phase. In polymeric membranes, water transport through the phase is usually characterized with higher diffusion coefficient compared to ion transport. The mechanism of water transport is different from that of ions—water is rather transported through pores of the membrane, whereas ions are moving through the polymeric phase. Water diffusion coefficient of order of 10^−6^ cm^2^/s in plasticized PVC membranes has been reported, regardless of used approach ranging from nuclear magnetic resonance (NMR), Fourier transform infrared spectroscopy (FT-IR), holography to electrochemical methods [111,112,113,114,115,116,117,118,119]. Clearly, water transport through the phase and potential accumulation is dependent on polymer used as matrix, but also transducer used in case of SC.

For SC-type sensors, water transported through ISM can reach the transducer layer. To minimize this risk, it is preferable to use lipophilic materials as the transducer layer. For more hydrophilic solid contacts accumulation of the liquid layer is clearly more probable. Possibility of formation of electrolyte ponds under the ISM phase has been highlighted especially for hydrophilic SC systems as poly(3,4-ethylenedioxythiophene) -poly(styrenesulfonate) [120]. The effect of the presence of an “aqueous layer” within the sensor can manifest itself predominantly in stability of potential readings over time [121,122]. The observed fluctuations of potential can be related to drying/rehydration of the SC layer if the sensor is stored dry/transferred to the sample [61], e.g., can result from just water transport through the phase. However, this process can be coupled with ion-exchange [121], as observed e.g., for ions reaching transducer systems [14], or systems able to bind primary ions within the transducer phase [58].

The presence of water at the back side of the membrane in the case of SC-type sensors can result in chemical change of the electrical lead, used to prepare e.g., simple disposable sensors. This effect has been reported for e.g., screen-printed electrodes used as support for potentiometric sensors—spontaneous chemical change of the printed electrode material, e.g., hydrolysis of inks components resulted in release of the lipophilic species from the SC phase to the membrane, leading to deterioration of sensor performance [72].

Contact of the ISM with the aqueous sample can result in release of membrane components to the solution. These effects have not been widely reported/quantified, nevertheless some of the components can results in toxicological issues, e.g., plasticizer. Recent results have confirmed that plasticizer leakage results in significant contents of these compounds in sample solution [123], reaching e.g., 20 ppm of 2-nitrophenyl octyl ether plasticizer found in sample solution after 12 h contact of this with membrane.

Leaching of ionophore [124] or ion-exchanger [125,126] from the membrane has been observed using an electrochemical approach, looking at change in electrochemical properties of the membrane. These studies clearly confirmed that contents of these components are changing during the sensor’s lifetime, this effect can be also related to e.g., absorption of lipophilic species on the membrane surface [124]. Leaching of ion-exchanger from the membrane phase was observed to affect the detection limit of ISM-based potentiometric sensors [127]. It is accepted that spontaneous, unwanted release of ionophore from the membrane limits sensor lifetime [128]. The effect is related to the charge of the ionophore, its lipophilicity and complexation constants [128]. To eliminate unwanted leakage of ionophore, strategies involving immobilization/ covalent attachment were proposed, helping also to prevent other processes occurring between ionophore molecules as e.g., dimerization [98,129,130,131,132]. On the other hand, mechanical instability of the sensor as such (e.g., detachment of phases in the case of SC) can also affect performance of potentiometric sensors [80,133,134], and a possible remedy to avoid this effect is covalent binding of sensor phases [135,136].

## 5. Conclusions

Ion-selective membranes’ composition constancy is at continuous risk through sensor construction, pretreatment and operation. Due to the different nature of materials and processes involved, various effects can be observed. More typically, these lead to deterioration of a sensor’s performance. Application of new materials to host the ionophore/ion-exchanger to serve as solid contact needs to take into account potential interaction of major and minor components of the used materials with ISM both in preparation and in operation of the sensor.
